# Cytochrome P450 2C19 Poor Metabolizer Phenotype in Treatment Resistant Depression: Treatment and Diagnostic Implications

**DOI:** 10.3389/fphar.2019.00083

**Published:** 2019-02-19

**Authors:** Marin Veldic, Ahmed T. Ahmed, Caren J. Blacker, Jennifer R. Geske, Joanna M. Biernacka, Kristin L. Borreggine, Katherine M. Moore, Miguel L. Prieto, Jennifer L. Vande Voort, Paul E. Croarkin, Astrid A. Hoberg, Simon Kung, Renato D. Alarcon, Nicola Keeth, Balwinder Singh, William V. Bobo, Mark A. Frye

**Affiliations:** ^1^Department of Psychiatry and Psychology, Mayo Clinic Depression Center, Mayo Clinic College of Medicine and Science, Rochester, MN, United States; ^2^Department of Health Sciences Research, Mayo Clinic College of Medicine and Science, Rochester, MN, United States; ^3^Departamento de Psiquiatría, Facultad de Medicina, Universidad de los Andes, Santiago, Chile; ^4^Servicio de Salud Mental, Clínica Universidad de los Andes, Santiago, Chile; ^5^Universidad Peruana Cayetano Heredia, Lima, Peru

**Keywords:** pharmacogenomics, cytochrome P450, *CYP2C19*, *SLC6A4*, bipolar disorder

## Abstract

**Background:** Pharmacogenomic testing, specifically for pharmacokinetic (PK) and pharmacodynamic (PD) genetic variation, may contribute to a better understanding of baseline genetic differences in patients seeking treatment for depression, which may further impact clinical antidepressant treatment recommendations. This study evaluated PK and PD genetic variation and the clinical use of such testing in treatment seeking patients with bipolar disorder (BP) and major depressive disorder (MDD) and history of multiple drug failures/treatment resistance.

**Methods:** Consecutive depressed patients evaluated at the Mayo Clinic Depression Center over a 10-year study time frame (2003–2013) were included in this retrospective analysis. Diagnoses of BP or MDD were confirmed using a semi-structured diagnostic interview. Clinical rating scales included the Hamilton Rating Scale for Depression (HRSD_24_), Generalized Anxiety Disorder 7-item scale (GAD-7), Patient Health Questionnaire-9 (PHQ-9), and Adverse Childhood Experiences (ACE) Questionnaire. Clinically selected patients underwent genotyping of cytochrome P450 *CYP2D6*/*CYP2C19* and the serotonin transporter *SLC6A4*. PK and PD differences and whether clinicians incorporated test results in providing recommendations were compared between the two patient groups.

**Results:** Of the 1795 patients, 167/523 (31.9%) with BP and 446/1272 (35.1%) with MDD were genotyped. Genotyped patients had significantly higher self-report measures of depression and anxiety compared to non-genotyped patients. There were significantly more *CYP2C19* poor metabolizer (PM) phenotypes in BP (9.3%) vs. MDD patients (1.7%, *p* = 0.003); among participants with an S-allele, the rate of *CYP2C19* PM phenotype was even higher in the BP (9.8%) vs. MDD (0.6%, *p* = 0.003). There was a significant difference in the distribution of *SLC6A4* genotypes between BP (*l/l* = 28.1%, *s/l* = 59.3%, *s/s* = 12.6%) and MDD (*l/l* = 31.4%, *s/l* = 46.1%, *s/s* = 22.7%) patients (*p* < 0.01).

**Conclusion:** There may be underlying pharmacogenomic differences in treatment seeking depressed patients that potentially have impact on serum levels of *CYP2C19* metabolized antidepressants (i.e., citalopram / escitalopram) contributing to rates of efficacy vs. side effect burden with additional potential risk of antidepressant response vs. induced mania. The evidence for utilizing pharmacogenomics-guided therapy in MDD and BP is still developing with a much needed focus on drug safety, side effect burden, and treatment adherence.

## Introduction

In 2018, the World Health Organization (WHO) identified major depressive disorder (MDD) and bipolar disorder (BP) as leading causes of disability worldwide, negatively impacting over 360 million people (Whiteford et al., 2013; Vos et al., 2015; Ferrari et al., 2016; World Health Organization, 2018). While genetic factors are thought to contribute 59–85% to BP risk (McGuffin et al., 2003; Lichtenstein et al., 2009), and 31–42% to MDD risk (Sullivan et al., 2000) or shared genetic risk related to overlapping symptoms of bipolar and major depressive disorder (Lee et al., 2013; Doherty and Owen, 2014), there is less systematic research focused on pharmacokinetic (PK) and/or pharmacodynamic (PD) genetic variation in these two distinct patient groups. This may be of potential interest recognizing marked differences in rates of antidepressant response and antidepressant induced mania (AIM+) by diagnostic group (Frye et al., 2015).

Selective serotonin reuptake inhibitors (SSRIs) are now considered first-line treatment for MDD (Crismon et al., 1999; Anderson et al., 2008), but only an approximate 50% of patients with MDD achieve partial remission, and only 30% complete remission, with SSRI therapy (Rush et al., 2006). However, antidepressants have less evidence base in bipolar depression and may in fact contribute to mood destabilization (Frye, 2011; Sidor and Macqueen, 2011). PK and PD genetic variation (i.e., pharmacogenomics) may contribute to BP and MDD treatment-resistance (Porcelli et al., 2012).

The use of pharmacogenomics testing for mental illnesses therapy selection has increased (Drozda et al., 2014). Thus, the implementing of pharmacogenomics-guided recommendations may improve treatment outcomes for patient with treatment-resistance depression (Kung and Li, 2010; Rundell et al., 2011b). Several studies have shown improvement in antidepressant response rates associated with the use of pharmacogenomic testing in clinical settings (Hall-Flavin et al., 2012; Hall-Flavin et al., 2013; Winner et al., 2013) and a recent meta-analysis of four randomized controlled trials and two open label trials have shown the same results (Rosenblat et al., 2018). However, several other reports (Rosenblat et al., 2017; Zeier et al., 2018; Zubenko et al., 2018) have identified potential limitations of industry support and lack of blinding and control groups (Hall-Flavin et al., 2012, 2013).

The goal of this study was to assess the outcomes of PK and PD genetic variation in treatment seeking depressed patients with history of multiple drug failures/treatment resistance and assess results of genomic testing on subsequent treatment recommendations. We assessed the clinical value of pharmacogenomic testing examining the differences in psychometrics mean scores at baseline between genotyped and non-genotyped patients; and assessed the relationships between PK (*CYP2D6* and *CYP2C19*) or PD (*SLC6A4*) genetic variations, and MDD/BP severity scales in pharmacogenomically-tested vs. not tested patients.

## Materials and Methods

This study was approved by the Mayo Clinic Institutional Board. All participants provided written informed consent prior to enrollment, evaluation and blood draw in the Mayo Clinic Depression Center.

### Subjects

This was a naturalistic study. A consecutive sample of treatment-seeking adults (age 18–65) with a clinical diagnosis of MDD or BP, currently in a depressive episode, was recruited from the Mayo Clinic Depression Center between February 26, 2003 and March 27, 2013. Clinical diagnoses were confirmed by DSM-IV-TR Structured Clinical Diagnostic Interview (SCID). Inclusion criteria were based on patients who presented with long history of multiple drug failures or treatment resistance. Exclusion criteria were inability to provide written informed consent, other Axis I or II diagnoses that by clinical judgment were the main reason for seeking treatment, substance use disorder determined by clinical interview, and (+) drug screen (except nicotine and caffeine) were excluded. Data were abstracted from Electronic health record (EHR) by two reviewers (Caren J. Blacker and Kristin L. Borreggine). For data abstraction validation, 10% of the abstracted data was reviewed by Marin Veldic.

### Clinical Ratings

Psychometrics utilized in the consultation included the 24 item Hamilton Rating Scale for Depression (HRSD_24_) (Hamilton, 1960), Patient Health Questionnaire-9 (PHQ-9) (Spitzer et al., 1999), Generalized Anxiety Disorder 7 item scale (GAD-7) (Spitzer et al., 2006), and Adverse Childhood Experiences (ACE) questionnaire (Felitti et al., 1998); however, not all patients had all the scales completed at the time of consultation. Clinical demographics included age, gender, and treatment. The EHR data was reviewed to assess relevance of genotyping which was quantified as: (1) clinician providing genotype-guided recommendations (GGR), or (2) clinician providing treatment as usual (TAU), where genotyping was or was not acknowledged, but treatment was guided based on the discretion of the treating clinician.

### Genotyping

Subjects were evaluated for the clinical treatment decision impact of genetic testing for PK [cytochrome P450 *2D6* (*CYP2D6*) and *2C19* (*CYP2C19*)] and PD [serotonin transporter (*SLC6A4*)] genetic variation on treatment as usual in MDD or BP depressed patients. Testing was completed either with the AssureX Health GeneSight^®^ platform or individual testing of PK or PD genes by Mayo Medical Laboratory. *CYP2D6* phenotypes were defined pharmacokinetically as extensive metabolizer (EM), intermediate metabolizer (IM), poor metabolizer (PM), or ultrarapid metabolizer (URM). *CYP2C19* phenotypes were defined pharmacokinetically as EM, IM, or PM. Detailed *CYP2D6* and *CYP2C19* allele variants are showed in Supplementary Table S1. *SLC6A4*, phenotypes were defined as [long/long (*l/l*)], [short/long (*s/l*)], or [short/short (*s/s*)]. *SLC6A4* has other genetic variations that may be relevant for the analysis (Hu et al., 2006). As reviewed by Frye and colleagues, in addition to the L allele and the *SLC6A4* and the SNP rs2553, known to influence the association of the 5-HTTLPR alleles with expression of the *SLC6A4* gene, there is a second intron variable number of tandem repeats (VNTR) identified that would be of interest in subsequent analysis. However, these variants were not identified in earlier samples of this study (i.e., from 2003) (Frye et al., 2015). The simultaneous determination of the long and short form of *SLC6A4* was performed by polymerase chain reaction (PCR) amplification of the promoter region of 5-HTT followed by Haemophilus parainfluenzae II digestion of the resulting amplicon, as described by Wendland et al. (2006). *CYP2C19* and *CYP2D6* genotyping was performed on genomic DNA extracted from whole blood using the xTAG Assay for P450-2C19 v2, which incorporates multiplex PCR and multiplex allele-specific primer extension (ASPE) with Luminex Molecular Diagnostics’ proprietary Universal Tag sorting system on the Luminex 100 xMAP platform. Detailed genotyping laboratory methodology is highlighted in our previous work (Mrazek et al., 2009; Mrazek et al., 2011; Frye et al., 2015).

### Statistical Analysis

Means and standard deviations are presented for continuous variables, and were compared by genotyping status and recommendation group using *t*-test or Wilcoxon Rank Sum tests. Chi-squared test and Fisher’s exact test were used to describe the differences in proportions between the genotyping status and recommendation group. The level of statistical significance was set at *p* < 0.05 (two-sided).

## Results

### Patient Characteristics

Using the SCID, 523 of the 1795 patients were diagnosed with BP and 1272 were diagnosed with MDD. 167/523 (31.9%) with BP and 446/1272 (35.1%) with MDD were genotyped. 317 subjects (18%) and 510 subjects (28%) underwent *CYP2D6/CYP2C19* and *SLC6A4* genotyping, respectively. Genotyped patients were less prescribed antidepressants (*p* = 0.009) versus other medication classes, and had significantly higher measures of self-reported anxiety (GAD-7 = 12.9 (5.6), *p <* 0.016) and depression (PHQ-9 = 18 (6.1), *p <* 0.001) in comparison to non-genotyped patients (Table 1). PK and PD genotype-guided recommendations were associated with significantly higher measures of anxiety and depression [(GAD-7 = 13.2 (5.6), *p =* 0.02) and (PHQ-9 = 18.1 (6.1), *p =* 0.005), (GAD-7 = 13.2 (5.63), *p =* 0.009) and (PHQ-9 = 18.0 (6.1), *p <* 0.001), respectively] (Table 2). There was no significant association between different genotypes and the measures of anxiety and depression.

**Table 1:** Demographics and Clinical Characteristics of Patients with Genotyped *vs.* Not Genotyped.

	Genotyped (*n* = 613)	Not Genotyped (*n* = 1182)	*p*
**Demographic**			
Female	66.7%	61.6%	0.032
Age, years	43.3 (12.8)	42.0 (14.2)	< 0.001
MDD	72.8%	69.9%	0.202
**Race/Ethnicity**			
White (Caucasian)	89.2%	89.8%	0.282
Black/African American	1.1%	0.7%	
Asian	0.7%	1.8%	
American Indian/Alaskan Native	0.7%	0.3%	
Native Hawaiian/Pacific Islander	0.2%	0.1%	
Others/ Unknown	8.2%	7.3%	
**Rating scales**			
HRSD_24_	31.6 (8.5)	30.3 (9)	0.671
PHQ-9	18 (6.1)	15.5 (6.8)	< 0.001
GAD 7	12.9 (5.6)	10.4 (6.2)	< 0.016
ACE	2.0 (2.3)	2.2 (2.2)	0.412
**Medications**			
Mood stabilizers	12.4%	11.6%	0.617
Benzodiazepines	24.8%	24.1%	0.749
Antidepressants	33.1%	39.3%	0.009
Stimulants	4.7%	3.1%	0.093


*Values are expressed as Mean (SD) unless otherwise indicated; MDD, major depressive disorder; HRSD_*24*_, 24 item Hamilton Rating Scale for Depression; PHQ-9, item scale Patient Health Questionnaire-9; GAD 7, Generalized Anxiety Disorder 7; ACE, Adverse Childhood Experiences score. *p*-value, Chi-squared test.*

**Table 2:** Demographics and Clinical Characteristics of Patients with Genotype-Guided Recommendations (GGR) *vs.* Treatment as usual (TAU).

	*CYP2D6* / *CYP2C19*			*SLC6A4*		
	GGR (*n* = 317)	TAU (*n* = 1478)	*p*	GGR (*n* = 510)	TAU(*n* = 1285)	*p*
**Demographic**						
Female	63.4%	63.4%	0.979	66.3%	62.2%	0.104
Age, years	44.2 (13.1)	42.1 (13.9)	0.152	43.4 (12.8)	42.1 (14.1)	0.003
MDD	76.3%	69.7%	0.018	73.5%	69.8%	0.117
**Rating scales**						
HRSD_24_	32.4 (8.7)	30.6 (8.9)	0.867	32.2 (8.3)	30.2 (9.1)	0.240
PHQ-9	18.1 (6.1)	15.8 (6.7)	0.005	18.0 (6.1)	15.6 (6.8)	< 0.001
GAD 7	13.2 (5.6)	10.5 (6.2)	0.02	13.2 (5.63)	10.4 (6.2)	0.009
ACE	2.0 (2.3)	2.2 (2.2)	0.648	2.0 (2.2)	2.2 (2.2)	0.720
**Medications**						
Mood stabilizers	12.3%	11.8%	0.791	12.9%	11.4%	0.375
Benzodiazepines	28.7%	23.4%	0.046	26.5%	23.5%	0.186
Antidepressants	33.8%	38.0%	0.160	35.1%	38.1%	0.242
Stimulants	5.1%	3.4%	0.153	5.1%	3.1%	0.044


*Values are expressed as SD, mean unless otherwise indicated; MDD, major depressive disorder; GGR, Genotype-Guided Recommendations; TAU, treatment as usual. HRSD_*24*_, 24 item Hamilton Rating Scale for Depression; PHQ-9, item scale Patient Health Questionnaire-9; GAD 7, Generalized Anxiety Disorder 7; ACE, Adverse Childhood Experiences score. *p*-value, Chi-squared test.*

### CYP2C19

The pharmacogenomic profiles of *CYP2C19* were: PM (3.5%), IM (27.4%), and EM (69.1%). There was a higher rate of *CYP2C19* poor metabolizer phenotype in BP (9.3%) vs. MDD patients (1.7%, *p* = 0.003) (Table 3). Among those participants with an S-allele, the rate of *CYP2C19* PM phenotype was even higher in the BP (9.8%) vs. MDD (0.6%, *p* = 0.003). There was no significant difference in distribution of treatment guided recommendations groups between *CYP2C19* phenotypes [EM (GGR = 68.85%, TAU = 69.49%), IM (GGR = 27.05%, TAU = 28.81%), PM (GGR = 4.10%, TAU = 1.69%), (*p =* 0.67)] (Table 3).

**Table 3:** Phenotype results by diagnose and Genotype-Guided Recommendations (GGR) *vs.* Treatment as usual (TAU).

Gene	Phenotype	MDD	BP	*p*	Phenotype	GGR	TAU	*p*
*CYP2D6*	PM (*n* = 45)	13.2 %	17.3%	0.41	PM (*n* = 42)	16.0%	5.1%	0.087
	IM/EM (*n* = 242)	78.1 %	70.7%		IM/EM (*n* = 232)	75.0%	83.1%	
	URM (*n* = 30)	8.7 %	12.0%		URM (*n* = 29)	9.0%	11.9%	
*CYP2C19*	PM (*n* = 11)	1.7%	9.3%	**0.003**	PM (*n* = 11)	4.1%	1.7%	0.67
	IM (*n* = 87)	29.3%	21.3%		IM (*n* = 83)	27.1%	28.8%	
	EM (*n* = 219)	69.0%	69.3%		EM (*n* = 209)	68.9%	69.5%	
*SLC6A4*	*s/s* (*n* = 102)	22.7%	12.6%	**0.012**	*s/s* (*n* = 96)	20.9%	15.9%	0.13
	*s/l* (*n* = 253)	46.1%	59.3%		*s/l* (*n* = 246)	47.2%	32.1%	
	*l/l* (*n* = 155)	31.2%	28.2%		*l/l* (*n* = 150)	31.9%	26.8%	


*MDD, major depressive disorder; BP, bipolar depression. GGR, Genotype-Guided Recommendations; TAU, treatment as usual. PM, poor metabolizer; IM/EM, intermediate metabolizer/extensive metabolizer; URM, ultra-rapid metabolizer; l, long; s, short allele variants. *p-*value, Chi-squared test.*

### CYP2D6

The pharmacogenomic profiles of *CYP2D6* were: IM/EM (76.3%), PM (14.2%), and URM (9.5%). There was no significant difference in distribution of *CYP2D6* phenotypes by diagnosis (*p =* 0.41) (Table 2). There was no significant difference in distribution of treatment guided recommendations groups between *CYP2D6* phenotypes [PM (GGR = 16%, TAU = 5.1%), EM/IM (GGR = 75%, TAU = 83.1%), URM (GGR = 9%, TAU = 11.9%), (*p =* 0.087)] (Table 3).

### SLC6A4

The pharmacogenomic profiles of *SLC6A4* were: *l/l* (30.4%), *s/l* (49.6%), and *s/s* (20.0%). There was a statistically significant difference in distribution of *SLC6A4* genotypes between BP (*l/l* = 28.2%, *s/l* = 59.3%, and *s/s* = 12.6%) and MDD (*l/l* = 31.2%, *s/l* = 46.1%, and *s/s* = 22.7%) patients (*p =* 0.012) (Table 3). Among S-allele carries, in comparison to MDD patients, there was a significantly higher rate of BP patients with PM in either *CYP2D6* or *CYP2C19*. There was no significant difference in distribution of treatment guided recommendations groups between *SLC6A4* phenotypes [*l/l* (GGR = 31.9%, TAU = 26.8%), *s/l* (GGR = 47.2%, TAU = 32.1%), and *s/s* (GGR = 20.9%, TAU = 15.9%) (*p =* 0.13)] (Table 3).

## Discussion

This study assessed the relationship between symptom severity, demographics, and pharmacokinetic / pharmacodynamics genetic variation among diagnostic mood disorder subgroups. There was a significant difference in *CYP2C19* and *SLC6A4* PK and PD phenotype distribution between BP and MDD patients with history of multiple drug failures/treatment resistance. Specifically, there were significantly higher rates of *CYP2C19* PM in BP patients in comparison to MDD patients; among those participants with an S-allele, the rate of *CYP2C19* PM phenotype was more than 10X higher in the BP vs. MDD.

The clinical implications of *CYP2C19* and serotonin transporter genetic variation are not fully understood. It is known, however, that poor metabolizer phenotype is associated with high blood levels and increased risk of side effects. As suggested by the Clinical Pharmacogenetics Implementation Consortium (CPIC) Guideline and Nassan et al. (2016), individuals on citalopram / escitalopram with *CYP2C19* PM phenotype should reduce dose by 50% and /or use an alternative antidepressant (Hicks et al., 2015). There has been little investigation as to metabolizer status, blood level, and risk of antidepressant induced mania in either bipolar or unipolar depression. There is evidence to suggest that the *s/s* genotype is associated with increased antidepressant side-effects, including antidepressant-induced mania (Frye et al., 2015); further studies should investigate risk of Antidepressant Induced Mania (AIM+) as a function of PK-PD interaction as is being done with other antidepressant pharmacogenomic antidepressant analyses (Ahmed et al., 2018). The genotype-guided recommendations of *CYP2D6*, *CYP2C19*, and *SLC6A4* were associated with significantly higher measures of anxiety and depression in comparison to treatment as usual. Like Rundell et al., 2011a, our study has found significantly higher baseline self-reported scores of depression in GGR individuals’ possible indicative of increase symptom burden and greater treatment resistance.

The results of *CYP2D6 / CYP2C19* genotyping were more commonly used to make treatment recommendations in MDD than in BP. This study is limited by it cross sectional design with no longitudinal mood outcome data based on GGT vs. TAU. However, there is increasing interest and investigation identifying PK *CYP2D6* and *CYP2C19* genetic variants associated with clinical response to several SSRIs (Tsai et al., 2010; Mrazek et al., 2011; Gressier et al., 2015; Hicks et al., 2015). Several studies have investigated GGR vs. TAU in treatment of MDD patients. However, none have included BP patients. Studying ACE score in relationship with *SLC6A4* S-allele and depression severity is also important, as there are gene and environment interactions (Caspi et al., 2003).

### Limitations

The decision to genotype was based on clinical factors and not pre-determined systematic criteria. Typically, patients who received genotyping might also have been self-selected and more interested in receiving it. Thus, there is inherent selection bias affecting the comparison between the two diagnostic groups. Even though the sample size was large, given the lower prevalence of *CYP2C19* PM and *SLC6A4* S-allele, the final number of patients with these findings were (*n* = 6) and, ideally, the initial sample size should be larger. This study did not have systematic follow-up to look at outcome measures of efficacy and side effects/tolerability based on these recommendations; these are important prospective studies to complete and such studies are currently underway. Our outcomes data have lacked the statistical power to accurately analyze the ancestry data; due to 89% of our population being white Caucasians, this may have affected the interpretation of our findings, this study was conducted in a clinical setting with a naturalistic study design, and is lacking standard criteria for the selection of patients for pharmacogenetic testing (Gelernter et al., 1997; Mrazek et al., 2009; Strom et al., 2012). Although, this type of design has the advantage of mimicking “real life” clinical practice, it has significant limitations when it comes to controlling for confounding. This is an issue that needs to be addressed in the future through longitudinal prospective studies with systematic genetic screening. Finally, our sample data was deficient of medication blood levels, which would clarify some of the study findings.

## Conclusion

There may be underlying pharmacogenomic differences in treatment seeking depressed patients that potentially have impact on serum levels of *CYP2C19* metabolized antidepressants (i.e., citalopram / escitalopram) contributing to rates of efficacy vs. side effect burden with additional potential risk of antidepressant response vs. induced mania. The evidence for utilizing pharmacogenomics-guided therapy in MDD and BP is still developing with a much needed focus on drug safety, side effect burden, and treatment adherence. Future work is essential; scientific and logistic barriers still exist before there can be widespread implementation of clinical genomics. Genomic science has a profound potential to individualize the drug therapy for depression.

## Ethics Statement

This study was carried out in accordance with the recommendation of the Mayo Clinic Institutional Review Board with written informed consent from all subjects. All subjects gave written informed consent in accordance with the Declaration of Helsinki. The protocol was approved by the Mayo Clinic Institutional Review Board.

## Author Contributions

ATA and JRG performed all the data analysis. CJB, KLB, and MV contributed to the acquisition of data. JMB assisted with data analysis and interpretation of findings. All co-authors provided critical revision of the manuscript for important intellectual content. MV and MAF were responsible for the study concept and design. MV an ATA drafted the manuscript. All authors critically reviewed content and approved the final version for publication.

## Conflict of Interest Statement

Mayo Clinic has a financial interest in AssureX Health and the technology referenced in this abstract.
