# Supplementary material for: Cytochrome P450 2C19 Poor Metabolizer Phenotype in Treatment Resistant Depression: Treatment and Diagnostic Implications
Source: Front Pharmacol. 2019 Feb 19;10:83. doi: 10.3389/fphar.2019.00083 (PMC6389687; doi:10.3389/fphar.2019.00083)
Supplement: Supplementary file 1 [file Table_1.docx]

| **Supplemental table 1: CYP2D6 and CYP2C19 allele variants** | | | |
| --- | --- | --- | --- |
| CYP2D6  (Genotype) | % | CYP2C19 (Genotype) | % |
| *1/*2A | 17.35 | *1/*1 | 69.09 |
| *1/*1 | 14.51 | *1/*2 | 25.55 |
| *1/*4 | 14.2 | *2/*2 | 3.15 |
| *2A/*2A | 7.57 | *1/*8 | 1.26 |
| *2A/*4 | 6.94 | *1/*8 | 1.26 |
| *1/*41,*4/*4, *4/*41 | 4.73 | *1/*4,*2/*1,*2/*3 | 0.32 |
| *2A/*41 | 2.84 |  |  |
| *1/*9 | 2.52 |  |  |
| *1/*3, *1/*5 | 2.21 |  |  |
| *1/*10 | 1.89 |  |  |
| *1/*2,*2A/*6, *4/*10, *9/*41 | 1.26 |  |  |
| *2A/*5,*3/*4, *41/*41 | 0.95 |  |  |
| *2A/*10,*2A/*3, *2A/*9,*4/*6, *5/*41 | 0.63 |  |  |
| *1/*6,*10/*17, *2/*17,*2/*41, *2A/*17,*3/*5, *4/*9, *5/*10 | 0.32 |  |  |

Base on the Human Cytochrome P450 (CYP) Allele Nomenclature

Committee website (Sim and Ingelman-Sundberg, 2010).

|  |
| --- |
